# *BRCA1* and *BRCA2* genes mutations among high risk breast cancer patients in Jordan

**DOI:** 10.1038/s41598-020-74250-2

**Published:** 2020-10-16

**Authors:** Munir Abu-Helalah, Belal Azab, Rasmi Mubaidin, Dema Ali, Hanan Jafar, Hussam Alshraideh, Nizar Drou, Abdalla Awidi

**Affiliations:** 1 Department of Public Health, Faculty of Medicine, Mutah University, Karak, Jordan; 2 Faculty of Medicine, Al-Faisal University, Riyadh, Kingdom of Saudi Arabia; 3 Cell Therapy Center, The University of Jordan, Amman, 11942 Jordan; 4 Department of Pathology, School of Medicine, The University of Jordan, Amman, Jordan; 5 Radiation Therapy Department, Al-Bashir Hospital, Ministry of Health, Amman, Jordan; 6 Department of Anatomy and Histology, School of Medicine, The University of Jordan, Amman, Jordan; 7 Industrial Engineering Department, University of Science and Technology, Irbid, Jordan; 8 Industrial Engineering Department, American University of Sharjah, Sharjah, UAE; 9 NYU Abu Dhabi Center for Genomics and System Biology, Abu Dhabi, UAE; 10 Department of Medicine, School of Medicine, The University of Jordan, Amman, Jordan

**Keywords:** Cancer, Computational biology and bioinformatics, Genetics, Molecular biology

## Abstract

Familial breast cancer is estimated to account for 15–20% of all cases of breast cancer. Surveillance for familial breast cancer is well-established world-wide. However, this service does not exist in Jordan, due to the scarcity of information with regard to the genetic profiling of these patients, and therefore lack of recommendations for policy-makers. As such, patients with very strong family history of breast or ovarian cancers are not screened routinely; leading to preventable delay in diagnosis. Whole coding sequencing for *BCRA1*/*BCRA2* using next-generation sequencing (NGS)/Ion PGM System was performed. Sanger sequencing were then used to confirm the pathogenic variants detected by NGS. In this study, 192 breast cancer patients (and 8 ovarian cancer cases) were included. The prevalence of recurrent pathogenic mutations was 14.5%, while the prevalence of newly detected mutations was 3.5%. Two novel pathogenic mutations were identified in *BRCA2* genes. The common mutations in the Ashkenazi population used for screening may not apply in the Jordanian population, as previously reported mutations were not prevalent, and other new mutations were identified. These data will aid to establish a specific screening test for *BRCA 1*/*BRCA2* in the Jordanian population.

## Introduction

Breast cancer is the most common cancer in females worldwide contributing to 25.4% of the total number of new cancer cases among women in 2018. Worldwide, more than two million new cases are diagnosed each year and nearly 600,000 women die from breast cancer each year^1^. According to the Jordanian National Cancer Registry report of the year 2015, there were 1145 female breast cancer cases accounting for 20.6% of all newly-diagnosed cancer cases and 39.4% of the cancers in females^2^.

Breast cancer at an early age is more likely to be associated with an increased familial risk as hereditary predisposition is estimated to cause about 5–10% of all breast cancers^3^. Nearly 50% of all hereditary breast cancer cases are due to germline mutations in the *BRCA1*/*BRCA2* genes and are associated with early-onset breast cancer^4^. The cumulative risk estimates for developing breast cancer by age 80 are 72% for *BRCA1* carriers and 69% for *BRCA2* carriers^4^.

Nearly 1650 mutations and sequence variants have been reported in the *BRCA1* gene and 1730 in *BRCA2*^5^. *BRCA1* and *BRCA2* are both tumor suppressor genes that play a role in DNA repair. The *BRCA1* gene plays also a role in checkpoint control. Mutations in either gene are associated with an autosomal dominant inherited form of breast cancer^6,7^.

Several studies in the Arab world were done to identify the mutations in *BRCA1*/*BRCA2* genes and to assess prevalence of known mutations^8–12^. The main limitations for the above studies have been the small number of participants and the uncontrolled selection criteria. A recent study conducted in Jordan showed that *BRCA1/BRCA2* variants are common among Jordanian breast cancer patients as 27% of high-risk breast cancer patients had pathogenic or likely pathogenic variants in *BRCA1* or *BRCA2* genes^9^.

Recently, a comprehensive assessment of the mutational spectrum of *BRCA1*/*BRCA2* in the Middle East, North Africa, and Southern Europe was published^13^. Laitamn et al. reported recurring pathogenic sequence variants (PSVs) identifying 232 PSVs and 239 PSVs in *BRCA1* and *BRCA2*, respectively. This comprehensive data will lead to directed testing of high-risk subpopulations, and therefore earlier diagnosis and superior management leading to better outcomes. However, there is no founder mutation(s) in *BRCA1*/*BRCA2* in the Jordanian population that could be used for early genetic screening*.*

In this study, the authors evaluated the potential contribution and the frequency of mutations in the *BCRA1*/*BCRA2* genes in selected Jordanian patients with breast and ovarian cancers, identified based on NICE criteria for high risk of familial cancer, through performing whole coding sequence for *BCRA1*/ *BCRA2* with the purpose of providing baseline data for establishment of familial cancer services in Jordan.

## Materials and methods

### Ethical approval and consent to participate

The study was approved by the Institutional Review Boards and Ethics Committees at Mutah University and the Central Ethics Committee at the Ministry of Health, Jordan. All included patients were informed about the objective and protocol of the study and gave written informed consent in accordance with the Declaration of Helsinki prior to undergoing any procedures.

### Study population

This prospective cross-sectional study was conducted et al. Bashir Hospital, Amman, Jordan. All Jordanian breast cancer patients referred to this hospital during the study period were under surveillance for familial breast or ovarian cancer according to a uniform selection criterion (see below).The U.K. National Institute for Health and Care Excellence (NICE), November 2013 Clinical guidelines for identification of patients with high risk for familial breast/ovarian cancer who required referral for testing in a specialist genetics clinic were used for identifying study participants and labelling them as ‘high risk for familial cancer^14^.

Patients files were reviewed for clinical and pathology data such as stage, grade, site, laterality, oestrogen receptor status, HER2 (or HER2 neu) positive status, etc.

### Sample collection and genomic DNA isolation

Peripheral blood samples were drawn from study participants (192 breast cancer patients and 8 ovarian cancer cases) and sent for analysis at the Cell Therapy Centre at Jordan University. Genomic DNA was extracted from 5 ml EDTA tubes using QIAprep Spin Miniprep Kit according to manufacturer's recommendations, and quantified using Nano Drop Spectrophotometer (Thermo Fisher Scientific).

### Next-generation sequencing and variant analysis

#### *BRCA1*/*BRCA2* library preparation and Ion PGM next-generation sequencing

The coding exons and flanking intronic regions of the *BRCA1*/*BRCA2* genes were amplified using Ion AmpliSeq Library Kit 2.0 (Life Technologies) and Ion AmpliSeq *BRCA1*/*BRCA2* Panel (Life Technologies). The whole process was performed following the manufacturer’s instructions with minor modifications as previously described^15,16^. Each library was barcoded using Ion Xpress Barcode Adapters kit (Life Technologies) and its concentration was determined with Ion Library TaqMan Quantitation Kit (Life Technologies). For template preparation, emulsion PCR and enrichment were carried out using Ion OneTouch 2 system and the Ion OneTouch ES Instrument (Life Technologies) with Ion PGM Hi-Q View OT2 Kit (Life Technologies). An average of six prepared barcoded samples were loaded into a single Ion 316 Chip v2 (Life Technologies) per sequencing run. The sequencing reactions were performed in the Ion torrent PGM System (Life Technologies) using sequencing Ion PGM Hi_Q view sequencing kit (Life Technologies). The generated data on Ion PGM were processed through on Torrent Suite, version 5.0.4, running on a local server (Life Technologies). The VCF files were uploaded to Ion Reporter (Life Technologies) and annotated for single-nucleotide variants (SNV), insertions and deletions (indels), and splice site changes. Reads were visualized with the Integrative Genomics Viewer (IGV) and Alamut (Interactive Biosoftware, Rouen, France).

### Evaluation of the variants clinical significance

Variants that are clearly classified in the Breast Cancer Information Core (BIC) database, Human Gene Mutation Database Pro (HGMD pro) and ClinVar databases, and not previously described as pathogenic in the literature were evaluated based on the guidelines and standards for interpretation of sequence variants recommended by the American College of Medical Genetics and Genomics (ACMG)^17^.

### DNA Sanger sequencing

Sanger sequencing was performed for validating selected candidate variants identified by NGS. Primers flanking the candidate loci were designed using Primer 3.0 and synthesized by IDT, CA, USA (Supplementary table 1). PCR amplification was performed using Platinum PCR SuperMix (Invitrogen, CA, USA). Direct automated sequencing for the purified PCR products was performed on an ABI 3500 Capillary DNA Sequencer using the BigDye Terminator Cycle Sequencing kit (Applied Biosystems, CA, USA). The sequence chromatograms were visualized by Sequencing Analysis SeqA software (Applied Biosystems) and analyzed by Chromas Pro software (Technolysium Ltd., South Brisbane, Australia).

### Statistical analysis

Collected Data was analyzed using R Statistical Computing Software version 3.4.3 (R Foundation for Statistical Computing, Vienna, Austria). Descriptive statistics including means and standard deviations are reported for numerical patient characteristics while frequencies and percentages are reported for categorical characteristics. Predictors of *BRCA1* and *BRCA2* mutations were identified through multiple linear regression with stepwise selection. A significance level of 0.05 is used throughout the analysis.

## Results

### Participants

Of the 200 patients included in the study; 192 of them had breast cancer as the primary cancer site, while the remaining eight cases ovarian cancer as the primary site. 7.5% of them (15 patients) had bilateral breast cancer and 3% (6 patients) had recurrent cancer. The mean age of the study population was 43.6 ± 2.7 with an age interval of 22 to 70. Regarding clinical characteristics of participants (Table 1), 45% of patients were diagnosed at stage III or IV, while 32% of them were diagnosed at stage II. 26.5% had distant and 52.5% had regional metastasis at diagnosis. Large proportion (83%) of them had tumor size of 2 cm or more at time of diagnosis. HER2 receptor status was positive in 24.5% and negative in 64.5% of study participants, while the status was not reported for 3.5% of the cases (Table 1).

**Table 1:** Clinical characteristics of study participants.

Value	N	Raw %	Valid %	Cumulative %
**Primary site**				
Breast	192	96	96	96
Ovaries	8	4	4	100
**Site of cancer**				
Bilateral	15	7.5	7.5	7.5
Left	92	46	46	53.5
Right	93	46.5	46.5	100
**Stage at diagnosis for breast cancer**				
I	46	23	23	23
II	64	32	32	55
III	34	17	17	72
IV	56	28	28	100
**Metastasis**				
No	148	74	74	74
Yes	52	26	26	100
**Recurrence since baseline**				
No	194	97	97	97
Yes	6	3	3	100
**Laterality for breast cancer**				
Bi	16	8	8	8
Uni	184	92	92	100
**Tumor size at histological examination**				
(< 2 cm)	34	17	17	17
(> = 2 cm)	166	83	83	100
**Extent of disease**				
Distant	53	26.5	26.6	26.6
Local	41	20.5	20.6	47.2
Regional	105	52.5	52.8	100
**Oestrogen Receptors status**				
Negative	7	3.5	3.6	3.6
Positive	29	14.5	14.9	18.6
Unknown	158	79	81.4	100
**ER**				
Negative	37	18.5	19.1	19.1
Positive	139	69.5	71.6	90.7
Unknown	18	9	9.3	100
**PR**				
Negative	46	23	23.7	23.7
Positive	133	66.5	68.6	92.3
Unknown	15	7.5	7.7	100
**Hert-Neu**				
Negative	129	64.5	66.8	66.8
Positive	49	24.5	25.4	92.2
Unknown	15	7.5	7.8	100
**E cadherin**				
Negative	18	9	9.3	9.3
Positive	16	8	8.2	17.5
Unknown	160	80	82.5	100

### Overall *BRCA1*/*BRCA2* mutations

The overall prevalence of recurrent and novel mutations (pathogenic and variants of uncertain significance (VUS)) in *BRCA1*/ *BRCA2* genes was 18% (Table 2). The prevalence of recurrent pathogenic mutations was 14.5%, while the prevalence of novel mutations (pathogenic and VUS) was 3.5%. Two confirmed novel pathogenic mutations were identified in *BRCA2* genes. Regarding site of mutation, 7.5% (15 patients) had pathogenic mutations in *BRCA1* gene and 7% (14 patients) had pathogenic mutations in *BRCA2* gene. Five VUS novel variants in *BRCA2* gene were identified in this study. Regarding previously reported VUS, this study detected 6 in *BRCA1* genes and 9 in *BRCA2* genes (Table 3, Supplementary table 2).

**Table 2:** Prevalence of mutations pathogenic and VUS mutations.

Category	Number of patients	Prevalence
**Recurrent mutations**		
BRCA1 Positive	15	7.50%
BRCA2 Positive	14	7.00%
BRCA1 or BRCA2 Positive	29	14.50%
**Possible (recurrent and novel) mutations**		
BRCA1 Positive	7	3.50%
BRCA2 Positive	14	7.00%
BRCA1 or BRCA2 Positive	21	10.50%
**Recurrent and novel (VUS and pathogenic) mutations**		
BRCA1 Positive	15	7.50%
BRCA2 Positive	21	10.50%
BRCA1 or BRCA2 Positive	36	18.00%

**Table 3:** Pathogenic BRCA1/*BRCA2* variants found in breast/ovarian cancer patients (n = 200).

Variant				Case Freq /Zygosity	Type of cancer	Mutation Database			dbSNP ID	Protein Prediction				MAFgnomAD (%)	Reference
Exon /Intron	HGVS cDNA	HGVS aa	Variant Effect			ClinVar	BIC	HGMD(Accession #)		SIFT	PolyPhen-2	Mutation Taster	CONDEL		
***BRCA1*: pathogenic variants**															
E18	c.5186C>A	p.Ala1729Glu	Missense	1 (0.5%) Het	Breast	Pathogenic	CI	DM CM950153	rs28897696	Deleterious	Probably damaging	Disease causing	Deleterious	0.0004	^21^
E18	c.5158C>T	p.Arg1720Trp	Missense	1 (0.5%) Het	Breast	Pathogenic	CI	DM CM041706	rs55770810	Deleterious	Probably damaging	Disease causing	Deleterious	0.002	^21^
E10	c.4065_4068delTCAA	p.Asn1355Lysfs*10	Frameshift	2 (1%) Het	Breast	Pathogenic	CI	DM CD941619	rs80357508	–	–	–	–	0.0008	^22^
E10	c.1224delA	p.Val409*	Nonsense	1 (0.5%) Het	Ovarian	Pathogenic	N/A	DM CD159317	rs879255320	–	–	–	–	N/A	^23^
E19	c.5224C>T	p.Gln1742*	Nonsense	1 (0.5%) Het	Ovarian	Pathogenic	N/A	DM CM1211123	N/A	–	–	–	–	N/A	^24^
E3	c.121C>T	p.His41Tyr	Missense	2 (1%)Het	Breast	N/A	N/A	DM CM1712699	N/A	Deleterious	Probably damaging	Disease causing	Deleterious	N/A	^25^
***BRCA2*: pathogenic variants**															
E10	c.2254_2257delGACT	p.Asp752Phefs*19	Frameshift	2 (1%) Het	Breast	Pathogenic	CI	DM CD023496	rs80359326	–	–	–	–	N/A	^28^
E11	c.5042_5043delTG	p.Val1681Glufs*7	Frameshift	1 (0.5%) Het	Breast	Pathogenic	CI	DM CD082063	rs80359478	–	–	–	–	N/A	^30^
E11	c.5351dupA	p.Asn1784Lysfs*3	Frameshift	1 (0.5%) Het	Breast	Pathogenic	CI	DM CI004026	rs80359508	–	–	–	–	N/A	^23^
E11	c.6634_6637delTGTT	p.Cys2212Leufs*16	Frameshift	2 (1%) Het	Breast	Pathogenic	N/A	DM CD077812	rs397507871	–	–	–	–	N/A	^24^
E11	c.6224_6225delAA	p.Lys2075Serfs*2	Frameshift	1 (0.5%) Het	Ovarian	N/A	N/A	N/A	N/A	–	–	–	–	N/A	Novel
E21	c.8696A>G	p.Gln2899Arg	Missense	1 (0.5%) Het	Breast	N/A	N/A	N/A	N/A	Deleterious	Probably damaging	Disease causing	Deleterious	N/A	Novel

*BIC* Breast Cancer Information Core, *HGMD* Human Gene Mutation Database, *MAF* Minor allele frequency, *E* Exon, *Het* Heterozygous, *Hom* Homozygous, *CI* Clinically Important, *DM* Disease causing mutation, *N/A* Not available.

**Figure 1:**
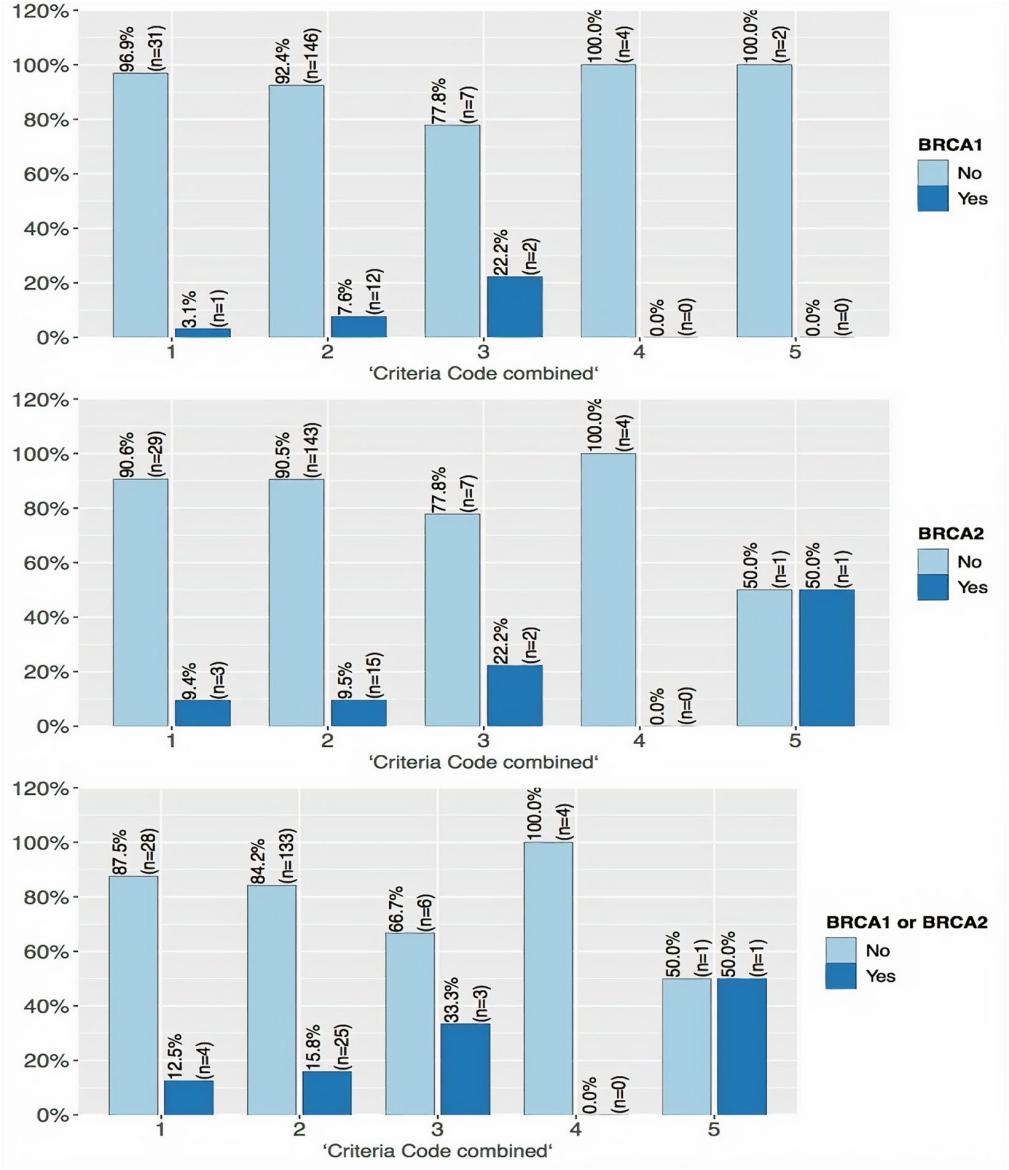
*BRCA1* and *BRCA2* variants versus inclusion criteria. Top *BRCA1* patients, middle *BRCA2* patients and bottom *BRCA1* and *BRCA2* patients. Criteria code combined: 1: Female breast cancer patients younger than the age of 35, 2: At least the following female breast cancers only in the family, 3: Families containing one relative with ovarian cancer at any age and, on the same side of the family, 4: Families affected by bilateral cancer (each breast cancer has the same count value as one relative, 5: Families containing male breast cancer at any age and, on the same side of the family.

Figure 1 shows mutations in *BRCA1*/*BRCA2* according to patients’ selection criteria. Participants with relatives with ovarian cancer had higher prevalence of mutations compared with other groups. Regression analysis results showed that E cadherin, PR, ER, oestrogen receptors status, laterality and recurrence since baseline were the statistically significant predictors of the presence of *BRCA1* mutations. On the other hand, E cadherin, laterality and recurrence since baseline were statistically significant predictors of *BRCA2* mutations.

### DNA sequencing and variant analysis

*BRCA1* and *BRCA2* coding regions and intronic boundaries were amplified. The PCR was performed using the 167 primer pairs. Ion Torrent PGM generated 150 bp average sequence reads length from the sequencer, with 455,768 bp mean target read, 97.5% Target base coverage at 100X, 96.36% uniformity of base coverage, 90.18% on targeted region with a 2,738X average base coverage depth of both genes.

**Figure 2:**
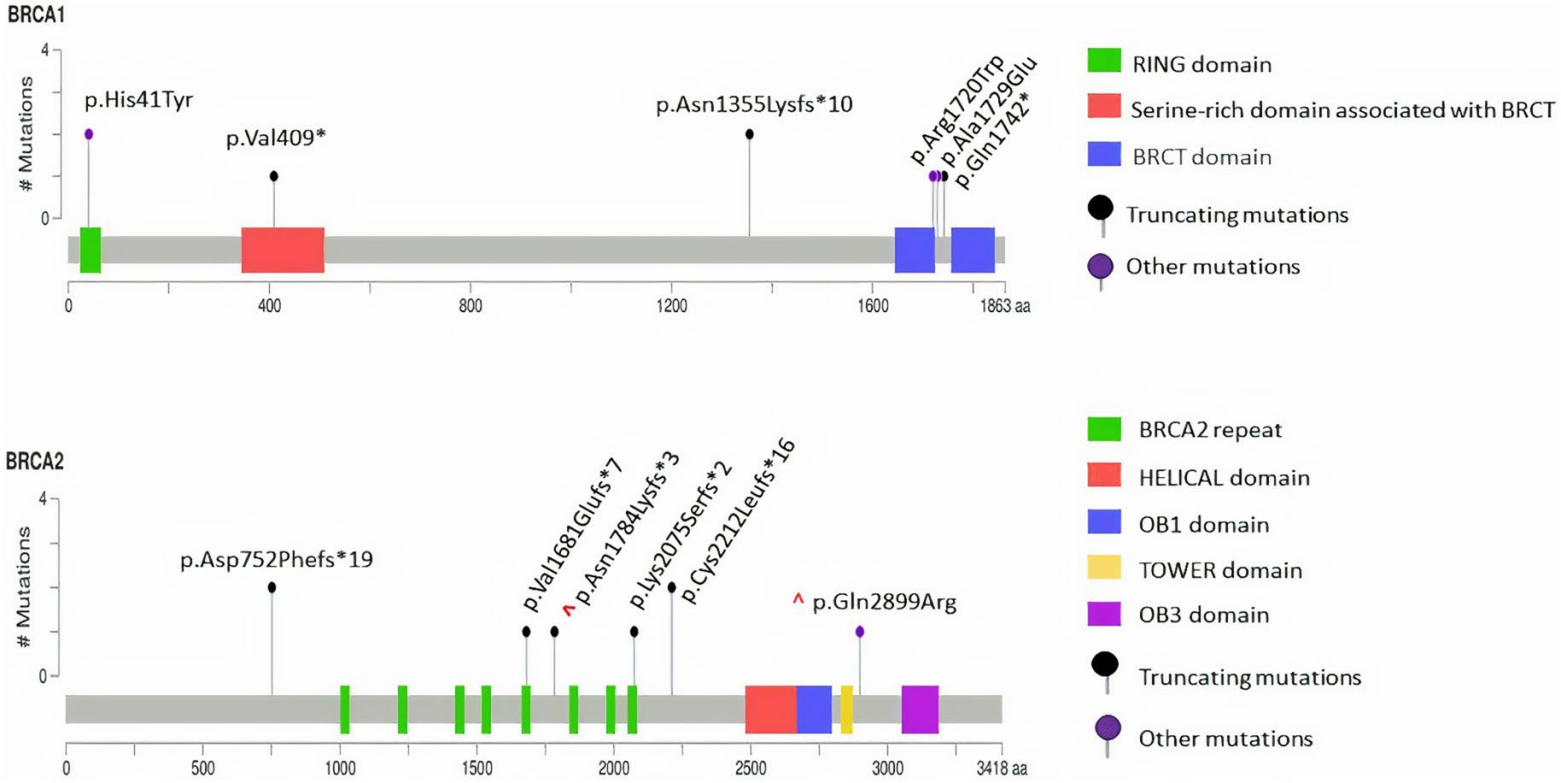
Schematic diagram of BRCA1 and BRCA2 proteins with the position of all identified pathogenic variants. Novel variants are marked with a caret (^).

*BRCA1*/*BRCA2* sequencing results revealed 37 different patients with pathogenic and VUS. Seven of those participants had novel variants that do not exist in the literature, diseases and population databases (Table 3, Supplementary table 2). As listed in Table 3, six *BRCA1* variants were pathogenic, two of them were nonsense, one frameshift and three missense variants. In addition, six *BRCA1* variants were VUS; four of them were missense variants, one VUS was synonymous, and another was a new splice site change (c.5396-6 T > C) that was present for the first time in our breast cancer patient (Fig. 2, Supplementary table 2). *BRCA2* germline variants are presented in Table 3. Two pathogenic *BRCA2* variants (p.K2075S fs*2; c.6224_6225delAA and p.Q2899R; c.8696A > G) were novel, they are neither registered in the disease nor the population databases. Those two novel pathogenic variants were further validated by Sanger sequencing (Supplementary Fig. 1 a-b). Four others recurrent *BRCA2* pathogenic variants were identified. They were registered in diseases databases but absent from the population databases gnomAD indicating they are rare variants (Fig. 2 and Table 3). The duplication variant in *BRCA2* (p.N1784K fs*3; c.5351dupA) was in a homopolymer region and therefore we validated it through Sanger sequencing (Supplementary Fig. 1c).We also found five distinct novel variant in *BRCA2* and classified them as VUS (p.S958R; c.2874 T > G, p.I1000L; c.2998A > C, p.H1525L; c.4574A > T, p. = (p.P2883P), c.8649A > T, p.T3204I; c.9611C > T). In addition, our cohort had nine variants that were classified as VUS (Supplementary table 2). However, the founder variants of *BRCA1* (c.185delAG, *BRCA1* c.5382insC), and *BRCA2* (c.6174delT) in the Ashkenazi Jewish population, were absent in the current cohort, indicating that *BRCA* variants vary among different populations^18^.

## Discussion

This study was set out to identify *BRCA1*/*BRCA2* mutations in patients at high risk of familial breast and ovarian cancer using a systematic approach. Our study showed a high prevalence of 18.0% for positive mutations in *BRCA1* (7.5%) or *BRCA2* (10.5%) genes. Two novel pathogenic mutations and five novel VUS variants were identified in this study, in addition to identifications of patients with recurrent pathogenic and VUS mutations. These figures are higher than recent data from neighboring Arab Peninsula of 16% or from other Asian countries like Japan of 7%^15–19^. This could be related to our inclusion criteria based on NICE guidelines for being at high risk of familial breast or ovarian cancers.

Out of 12 pathogenic variants detected in this study in both *BRCA1*/*BRCA2*, five pathogenic variants were reported previously among Caucasian, Western populations and Latin American. These variants including c.5186C > A,, c.4065_4068delTCAA in *BRCA1* and c.5042_5043delTG, c.5351dupA in *BRCA2*^20–24^*.*Additionally, three pathogenic variants were previously reported among Asia population. Both c.1224delA and c.5224C > T in *BRCA1* and c.6634_6637delTGTT in *BRCA2* were originally reported in Japan and China populations^25,26^. However, different recurrent pathogenic variants were previously reported among Arab population. The c.121C > T pathogenic variant in *BRCA1*and the c.2254_2257delGACT pathogenic variant in *BRCA2* were recently reported in Palestinian patients^27,28^. Additionally, the c.5158C > T pathogenic variant in *BRCA1* was previously reported in Morocco and Saudi Arabia^13^. Both c.6224_6225delAA and c.8696A > G variants in *BRCA2* have not been described in the BIC database and other databases including HGMD professional and ClinVar. Therefore, they are considered to be novel. It’s worth to mention that four variants in both *BRCA1* and *BRCA2* were common in more than one patient (Table 3)^29–32^.

Positive patients in our study (recurrent or novel mutations) were diagnosed at advanced stages of breast cancer and ovarian cancers, although they are at high risk of familial cancer. This preventable delay is expected to have a major negative impact on morbidity, morbidity, and cost of management. Such patients could have received preventive cancer treatment giving their very strong family history, should they have been tested properly in due time. Establishing the mutational profile in our region will allow directed testing of high-risk families, therefore increasing the benefit while reducing costs. This is an agreement with Laitman et al. who identified several highly-recurring mutations in the region^13^.

Our participants presented with breast cancer at an early age with a mean of 43.7 ± 2.7. Breast cancer at an early age is more likely to be associated with an increased familial risk, particularly in women with a germline *BRCA1* mutation^33^. In a study of women with breast cancer diagnosed before age 30, *BRCA1/BRCA2* and *TP53* mutations were found in about half who had strong family histories of breast cancer and in less than 10% of women with non-familial breast cancer^34^. A young age at diagnosis for breast cancer patients in Jordan and the Arab world suggests a high probability of familial predisposition. A systematic review on age at diagnosis for breast cancer in the Arab world revealed that average age was 48 (SD = 2.8), range 43–52, median 48.5 and mode 45. These figures are based on data from 28 articles that included years amongst the 7455 patients. The percentage of patients that were younger than 50 years old was reported in 11 articles from 8 countries and included 5144 patients; 65.5% (SD = 11) who were less than 50 years old (range 49–78%, median = 66%). It is recommended that further research to be conducted among young breast and/or ovarian cancer patients in the Arab world to examine the prevalence of known mutations and to search for new potential pathogenic mutations.

Identification of mutations in *BRCA1*/*BRCA2* genes leading to familial breast cancer has had a major impact on prevention of breast and ovarian cancer through development of clinical practice guidelines for management and referral systems for women with a personal or family history of breast or ovarian cancer^35^. Results from some countries (e.g. U.K., France and Canada) and specific cities (Manchester, Marseilles, and Montreal) provided excellent examples of the clinical and economic effectiveness of these preventive services^36^. Management of patients start as early as the age of 25 with clear guidelines for referral and management^37^. In many developing countries, including Jordan, this approach has not been put in place^38^. It has been well-reported in previous studies that positive carriers for *BRCA1*/*BRCA2* have high cumulative risk estimates for developing breast cancer reaching 72% for *BRCA1* carriers and 69% for *BRCA2* carrier by age of 80^5^. These patients could undergo preventive measures following timely identification of mutations, reducing morbidity and mortality arising from breast and ovarian cancers.

## Conclusion

This study assessed the prevalence of reported mutations and identified novel mutations for *BRCA1*/*BRCA2* genes. Giving the high prevalence of positive mutations in *BRCA1*/*BRCA2* genes, it is essential to establish a service for familial breast and ovarian cancer service in Jordan and the MENA region, that includes genetic counselling and early genetic screening.

## Supplementary information

Supplementary Table S2

Supplementary Table S1

Supplementary Legend

Supplementary Figure S1

## Data Availability

All relevant data is available through this manuscript and additional files.
