# Supplementary material for: BRCA1 and BRCA2 genes mutations among high risk breast cancer patients in Jordan
Source: Sci Rep. 2020 Oct 16;10:17573. doi: 10.1038/s41598-020-74250-2 (PMC7568559; doi:10.1038/s41598-020-74250-2)
Supplement: Supplementary file 1 — Supplementary Table S2 [file 41598_2020_74250_MOESM1_ESM.docx]

***BRCA1* and *BRCA2* genes mutations among high risk breast cancer patients in Jordan**

Munir Abu-Helalah, MD. PhD^1,2^, Belal Azab, PhD^3,4^, Rasmi Mubaidin, MD^5^, Dema Ali, MSc^3^, Hanan Jafar, PhD^3,6,^ Hussam Alshraideh, PhD^7,8^, Nizar Drou, PhD^9^, Abdalla Awidi, MD. FRCP. FRCPath ^3,10^,*

| Supplementary table 2: Uncertain significance *BRCA1/BRCA2* variants found in breast/ovarian cancer patients (n = 200) | | | | | | | | | | | | | | |
| --- | --- | --- | --- | --- | --- | --- | --- | --- | --- | --- | --- | --- | --- | --- |
| Variant | | | | Case Freq  / Zygosity | Mutation Database | | | dbSNP ID | Protein Prediction | | | | MAF gnomAD (%) | Reference |
| Exon /Intron | HGVS cDNA NM_007300.3 | HGVS aa | Variant  Effect |  | ClinVar | BIC | HGMD (Accession #) |  | SIFT | PolyPhen-2 | Mutation Taster | CONDEL |  |  |
| *BRCA1*: Uncertain Significance Variants | | | | | | | | | | | | | |  |
| E10 | c.1618G>A | p.Glu540Lys | Missense | 1 (0.5%)  Het | VUS | N/A | N/A | rs730881471 | Tolerated | Benign | Disease  causing | Deleterious | N/A | ClinVar dbSNP |
| E10 | c.3367G>T | p.Asp1123Tyr | Missense | 1 (0.5%)  Het | VUS | unkonwn | N/A | rs80356867 | Deleterious | Probably damaging | Polymorphisim | Deleterious | 0.0008 | 1 |
| E16 | c.4793C>A | p.Ser1598Tyr | Missense | 1 (0.5%)  Het | VUS | N/A | N/A | rs273901741 | Deleterious | Probably damaging | Polymorphisim | Deleterious | 0.0004 | ClinVar dbSNP |
| E19 | c.5249T>A | p.Leu1750Gln | Missense | 2 (1%) Het | VUS | N/A | N/A | rs730881496 | Deleterious | Probably damaging | Disease  causing | Deleterious | N/A | ClinVar ,dbSNP |
| I 21 | c.5396-6T>C | p.? | Spilce site | 1 (0.5%)  Het | N/A | N/A | N/A | N/A | __ | __ | __ | __ | 0.0004 | N/A |
| *BRCA2*: Uncertain Significance Variants | | | | | | | | | | | | | |  |
| E11 | c.2874T>G | p.Ser958Arg | Missense | 1 (0.5%) Het | N/A | N/A | N/A | N/A | Deleterious | Benign | Polymorphisim | Neutral | N/A | Novel |
| E11 | c.2998A>C | p.Ile1000Leu | Missense | 1 (0.5%) Het | N/A | N/A | N/A | N/A | Tolerated | Benign | Polymorphisim | Neutral | N/A | Novel |
| E11 | c.4574A>T | p.His1525Leu | Missense | 1 (0.5%) Hom | N/A | N/A | N/A | N/A | Tolerated | Benign | Polymorphisim | Deleterious | N/A | Novel |
| E21 | c.8649A>T | p.= (p.Pro2883Pro) | Synonymous | 1 (0.5%) Het | N/A | N/A | N/A | N/A | __ | __ | __ | __ | N/A | Novel |
| E26 | c.9611C>T | p.Thr3204Ile | Missense | 1 (0.5%) Het | N/A | N/A | N/A | N/A | Deleterious | Possibly damaging | Polymorphisim | Neutral | N/A | Novel |
| E2 | c.62A>G | p.Lys21Arg | Missense | 1 (0.5%) Het | VUS | N/A | N/A | rs397507367 | Tolerated | Benign | Polymorphisim | Neutral | 0.002 | ClinVar dbSNP |
| E4 | c.266C>T | p.Pro89Leu | Missense | 1 (0.5%) Het | VUS | N/A | N/A | rs748609599 | Deleterious | Probably damaging | Polymorphisim | Neutral | 0.001 | ClinVar dbSNP |
| E11 | c.5870T>C | p.Ile1957Thr | Missense | 1 (0.5%) Het | VUS | N/A | N/A | rs587782320 | Deleterious | Benign | Polymorphisim | Neutral | 0.002 | ClinVar dbSNP |
| E15 | c.7534C>T | p.Leu2512Phe | Missense | 1 (0.5%) Het | VUS | unknown | DM? CM1618159 | rs80358980 | Tolerated | Probably damaging | Disease  causing | Deleterious | 0.002 | 2 |
| E15 | c.7462A>G | p.Arg2488Gly | Missense | 1 (0.5%) Het | VUS | N/A | N/A | rs746057464 | Deleterious | Probably  damaging | Disease  causing | Deleterious | N/A | ClinVar dbSNP |
| E25 | c.9286G>A | p.Glu3096Lys | Missense | 1 (0.5%) Het | VUS | N/A | N/A | rs80359199 | Tolerated | Probably damaging | Disease  causing | Deleterious | 0.0004 | ClinVar dbSNP |
| E25 | c.9364G>A | p.Ala3122Thr | Missense | 1 (0.5%) Het | VUS | N/A | N/A | rs587782313 | Tolerated | Probably damaging | Disease  causing | Deleterious | 0.0001 | ClinVar dbSNP |
| E27 | c.9875C>T | p.Pro3292Leu | Missense | 1 (0.5%) Het | VUS | unknown | DM? CM1313389 | rs56121817 | Deleterious | Probably damaging | Disease  causing | Neutral | 0.008 | 3 |
| E27 | c.10115C>G | p.Ala3372Gly | Missense | 1 (0.5%) Het | N/A | N/A | N/A | rs748237097 | Tolerated | Benign | Polymorphisim | Neutral | 0.002 | dbSNP |

BIC, Breast Cancer Information Core; HGMD, Human Gene Mutation Database; MAF, Minor allele frequency; E, Exon; I, Intron; Het, Heterozygous; CI, Clinically Important; VUS, variant of uncertain significance; DM, Disease causing mutation; N/A , Not available.

References:

1- Seymour, I. J. *et al.* Results of a population-based screening for hereditary breast cancer in a region of North-Central Italy: contribution of BRCA1/2 germ-line mutations. *Breast cancer research and treatment* **112**, 343–349 (2008).

2- Maistro, S. *et al.* Germline mutations in BRCA1 and BRCA2 in epithelial ovarian cancer patients in Brazil. *BMC cancer* **16**, 934 (2016).

3- Tram, E., Savas, S. & Ozcelik, H. Missense variants of uncertain significance (VUS) altering the phosphorylation patterns of BRCA1 and BRCA2. *PloS one* **8**, (2013)
