# Supplementary material for: BRCA1 and BRCA2 genes mutations among high risk breast cancer patients in Jordan
Source: Sci Rep. 2020 Oct 16;10:17573. doi: 10.1038/s41598-020-74250-2 (PMC7568559; doi:10.1038/s41598-020-74250-2)
Supplement: Supplementary file 2 — Supplementary Table S1 [file 41598_2020_74250_MOESM2_ESM.docx]

***BRCA1* and *BRCA2* genes mutations among high risk breast cancer patients in Jordan**

Munir Abu-Helalah, MD. PhD^1,2^, Belal Azab, PhD^3,4^, Rasmi Mubaidin, MD^5^, Dema Ali, MSc^3^, Hanan Jafar, PhD^3,6,^ Hussam Alshraideh, PhD^7,8^, Nizar Drou, PhD^9^, Abdalla Awidi, MD. FRCP. FRCPath ^3,10^,*

Supplementary table 1: Primer oligonucleotide sequences used in Sanger sequencing

| Gene symbol | Variant | Primer sequence (5' - 3') | Amplicon position (GRCh37/hg19) | | Amplicon size |
| --- | --- | --- | --- | --- | --- |
| *BRCA2* | c.6224_6225delAA | F: CGCAAGACAAGTGTTTTCTGA R: CACAGTGCTCTGGGTTTCTCT | | chr13:32914477-32914844 | 368bp |
| *BRCA2* | c.8696A>G | F: TGAACCCAGGAGACAGAGGT R: GAAAACATACCACCACACTCGT | | chr13:32950576-32951104 | 529bp |
| *BRCA2* | c.5351dupA | F: GCTGCCCCAAAGTGTAAAGA R: CACAAGTTCCTCAACGCAAA | | chr13:32913251-32913934 | 684bp |
| *BRCA1* | c.121C>T | F: CAGTTCCTGACACAGCAGACA R: GGAGTTGGATTTTTCGTTCTCA | | chr17:41267617-41267963 | 347bp |

F, Forward direction; R, Reverse direction; bp, base pair.
