# Supplementary material for: BRCA1 and BRCA2 genes mutations among high risk breast cancer patients in Jordan
Source: Sci Rep. 2020 Oct 16;10:17573. doi: 10.1038/s41598-020-74250-2 (PMC7568559; doi:10.1038/s41598-020-74250-2)
Supplement: Supplementary file 3 — Supplementary Legend [file 41598_2020_74250_MOESM3_ESM.docx]

**Supplementary Fig. 1** Identifications of deleterious variants in BRCA1 and BRCA2 by NGS (top) and validated by Sanger sequencing (bottom) as visualized by IGV software and ChromasPro software, respectively. In IGV, insertions are indicated by a purple I and deletions are indicated with a black dash. Blue arrows represent presence of variants in sanger sequencing, initially revealed by NGS. (a) AA deletion at position 6224_6225 of cDNA of BRCA2 in patient B144; (b) A substitution A to G at position 8696 of cDNA of BRCA2 in patient B37; (c) A insertion at position 5351 of cDNA of BRCA2 in patient B140; (d) A substitution C to T at position 121 of cDNA of BRCA1 in patient B21.
