# Supplementary figures and images for: BRCA1 and BRCA2 genes mutations among high risk breast cancer patients in Jordan
Source: Sci Rep. 2020 Oct 16;10:17573. doi: 10.1038/s41598-020-74250-2 (PMC7568559; doi:10.1038/s41598-020-74250-2)

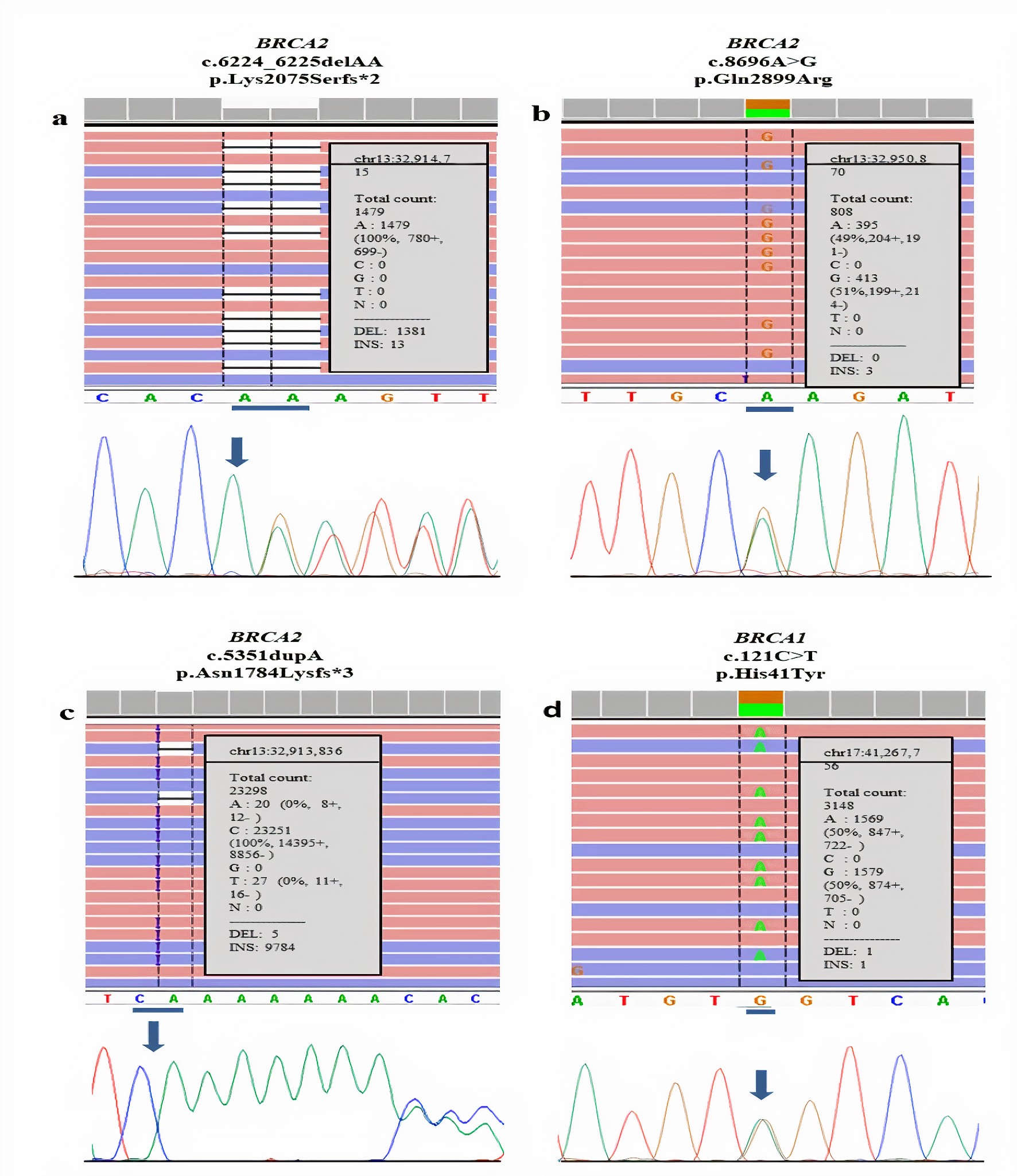

Supplement: Supplementary file 4 — Supplementary Figure S1 [file 41598_2020_74250_MOESM4_ESM.jpg]
